# Drug Repurposing of Metabolic Agents in Malignant Glioma

**DOI:** 10.3390/ijms19092768

**Published:** 2018-09-14

**Authors:** Corinna Seliger, Peter Hau

**Affiliations:** Department of Neurology and Wilhelm Sander-NeuroOncology Unit, University Hospital Regensburg, 93053 Regensburg, Germany; peter.hau@ukr.de

**Keywords:** glioma, glioblastoma, drug repurposing, metformin, statin, NSAR, disulfiram, lonidamine

## Abstract

Gliomas are highly invasive brain tumors with short patient survival. One major pathogenic factor is aberrant tumor metabolism, which may be targeted with different specific and unspecific agents. Drug repurposing is of increasing interest in glioma research. Drugs interfering with the patient’s metabolism may also influence glioma metabolism. In this review, we outline definitions and methods for drug repurposing. Furthermore, we give insights into important candidates for a metabolic drug repurposing, namely metformin, statins, non-steroidal anti-inflammatory drugs, disulfiram and lonidamine. Advantages and pitfalls of drug repurposing will finally be discussed.

## 1. Introduction

Gliomas are highly aggressive brain tumors with a poor patient prognosis [1]. They are classified using a recently revised multi-layer classification according to their histopathological characteristics, genetic markers and WHO grades I-IV, including information on the presence of a mutation in the isocitrate dehydrogenase gene (IDH) [2]. Glioblastomas (GBMs) are WHO grade IV gliomas with a high level of malignancy and are associated with a median overall survival ranging between 15 and 26 months for patients within clinical trials [3,4]. The standard of care for GBM includes postoperative combined radio-chemotherapy followed by chemotherapy with temozolomide alone [3]; depending on local practice-tumor-treating fields [5].

Patients without an IDH mutation [6], namely most patients with GBM, and patients with no methylation of the methylguanine-methyl transferase (MGMT) gene [7] and without a loss-of-heterozygosity at chromosome 1p19q (LOH 1p19q) [8] show worse clinical outcomes. Other important prognostic factors include the clinical performance score, age, extent of resection, comorbidities, primary and secondary therapies [3,9] and an early tumor progression [10] or persistent hyperglycemia as an important metabolic factor [11,12].

Beyond the standard of care as listed above, clinical trials are exploring targeted therapies, for instance, against the Vascular Endothelial Growth Factor (VEGF) [13], Epidermal Growth Factor [14] or Platelet Derived Growth Factor (PDGF) or their receptors [15]. Whether response to a targeted therapy can be predicted in vitro is still under discussion [16,17].

## 2. Tumor Metabolism and Glioma

The prognostic relevance of IDH mutations in glioma has drawn significant attention to tumor metabolism as a potential therapeutic target in recent times [6]. Long ago, in the 1920s, Otto Warburg already described the phenomenon of aerobic glycolysis in tumor cells [18]. According to the Warburg hypothesis, tumor cells tend to use glycolysis rather than oxidative phosphorylation to meet their energetic needs even in the presence of oxygen. Potential benefits for the tumor cells may be derived from the accumulation of intermediates for biosynthetic pathways, the faster provision of energy equivalents, the reduction of reactive oxygen species or the independence from intact mitochondria [19]. In addition, the final product of glycolysis, lactate, may also serve as an important pathogenic factor in glioma, for example by the activation of TGF-beta2 by thrombospondin [20] or by local immunosuppression [21]. Therefore, the research of Otto Warburg and his followers opened the door for a wide range of studies on the role of metabolic mechanisms and repurposing of metabolically active drugs in the therapy of glioma and beyond.

## 3. Definition and Principle of Drug Repurposing

Labels drug repurposing and drug repositioning are frequently used synonymously and definitions vary [22]. Both terms stand for the use of a drug for another indication rather than the one it was primarily investigated or approved for. Underlying reasons may be that new molecular findings allow for targeting by compounds that may already be part of drugs that are in clinical use for other diseases. Prominent examples are sildenafil [23,24] which is now used for erectile dysfunction and pulmonary hypertension, or thalidomide [25], which was initially used for sleeping disorders and nausea frequently leading to birth defects, but is now again used for multiple myeloma and leprosy.

There are different approaches to identifying drugs that may also serve for another indication. Pharmaco-epidemiological studies investigate the association between drug use and the incidence or progression of several tumors, including gliomas. Large databases such as the Clinical Practice Research Datalink (CPRD), a primary care database including over 8 million patients in the UK [26], or large population-based databases such as in Denmark [27] are used for this purpose. Other authors investigated pooled analyses of randomized clinical trials [28] or data collected within one or several institutions [11,12]. Often, research hypotheses have been generated out of such publications. However, epidemiological studies are often limited by the fact that they are retrospective in nature, which may give rise to several biases such as selection bias, confounding by indication or recall bias. Furthermore, in drug studies, immortal time biases, time-window biases, and time-lag biases are of major concern [29]. In addition, the mere observation of an association between drug use and incidence or progression of a tumor does not allow for the definition of biological effects or mechanisms. However, those may be then investigated in experimental analyses.

Another approach to identifying drugs for drug repurposing is the in silico drug repositioning. Thereby, bioinformatic algorithms serve to investigate the associations between drugs, targets and diseases. Different databases help analyze drug-target interactions on the genomic (NCBI Phenome-Genome Integrator [30], Genome-wide Association Studies Catalogue [31]) or proteomic level (Human Protein Atlas [32], human proteome map [33], proteomics database [34], UniProt Knowledgebase [35], canSAR protein annotation tool [36]) and in disease oriented databases (GeneCards [37], MalaCards [38], DisGeNET [39]) or databases focused on molecular targets (DrugBank [40], U.S. Federal Drug Administration Database and Drugs of New Indications database [41]).

Finally, activity-based drug repurposing is also an approach to identify new medications out of old drugs. In this case, actionable targets are detected for different diseases regardless of concurrent structural information. For example, ALK inhibitors such as crizotinib or ceritinib are used for different neoplasms with an over-activated ALK kinase (usually caused by an EML4-ALK rearrangement) such as neuroblastoma, non-small-cell lung carcinoma or anaplastic large cell lymphoma [42].

## 4. Metabolic Drug Repurposing in Glioma

Metabolic drugs are also under study for drug repurposing and include medications that are proposed to influence the whole patient’s lipid, protein or carbohydrate metabolism or only the metabolism within the neoplasm itself. A multitude of approved agents are under investigation for metabolic drug repurposing [43,44]. In this study, we will focus only on some of the most prominent candidates for a repurposing of metabolic drugs, namely on metformin, statins, non-steroidal anti-inflammatory drugs, disulfiram, and lonidamine.

### 4.1. Metformin

Metformin is a frequently used drug for the treatment of type 2 diabetes [45]. In addition to its known effects of lowering blood glucose, metformin was associated with a reduced risk of cancer [46]. Metformin was hypothesized to interfere with insulin-like growth factor signaling by lowering glucose and insulin levels, but also to directly inhibit complex 1 of the respiratory chain in tumor cells with subsequent activation of the adenosine monophosphate kinase (AMPK) and downstream inhibition of the mammalian target of rapamycin (mTOR) [47]. Other mTOR inhibitors are also under investigation as potential anti-glioma agents. There are currently over 30 of those medications in clinical trials (www.clinicaltrials.gov).

Several groups including ours investigated possible antineoplastic effects of metformin on glioma cells [48,49,50,51] and postulated inhibitory effects on glioma cell growth [48,52,53,54] and invasion [55], and on induction of apoptotic [48,52,54] or autophagic [50] cell death. Metformin was not found to inhibit transforming growth factor (TGF)-β_2_, as described for other entities [53], but a significant inhibition of the STAT3 signaling pathway could be observed [56].

In a large case-control analysis within the clinical practice research datalink (CPRD), we found that use of metformin was not significantly associated with a reduced risk of glioma (Odds Ratio (OR) for ≥30 vs. 0 prescriptions = 0.72; 95% Confidence Interval (CI) = 0.38–1.39), but there was a significant inverse association between having a history of diabetes and the incidence of glioma (OR = 0.74; 95% CI = 0.60–0.93 [57]). Possible explanations for the fact that metformin did not have an impact on glioma risk albeit significant effects on cell culture and tumor models may be the limited sample size of diabetic glioma patients on metformin in that study or intensified doses of metformin in laboratory investigations as compared to daily diabetes treatment. Many laboratory studies used doses of metformin around 10 mM, whereas 10 µM have been measured in the brain of diabetic patients [58]. Another possible explanation may be that metformin does not affect the development of glioma, but may have an influence on survival after glioma diagnosis.

Several studies analyzed how use of metformin influences progression of diabetic glioma patients. A small study based on 276 patients with GBM found a prolonged progression-free survival time in diabetic patients with metformin, which could not be recapitulated in multivariate analysis [11]. In a larger study, metformin was one of the most important predictors for better survival among 988 diabetic patients with GBM [59]. Interestingly, we found in a large study on 1093 patients that use of metformin was associated with improved Overall Survival (OS) and Progression Free Survival (PFS) of diabetic patients with WHO grade III (HR for OS = 0.30; 95% CI = 0.11–0.81), but not WHO grade IV glioma (HR = 0.83; 95% CI = 0.57–1.20) [60]. Patients with WHO grade III glioma have a higher incidence of IDH-mutations compared to patients with primary GBM. Potentially, patients with IDH-mutated gliomas are more susceptible to metformin due to a higher metabolic vulnerability [61].

Several randomized clinical trials evaluate the addition of metformin to adjuvant treatment of glioma (NCT02496741, NCT02149459, NCT02040376, NCT02780024, NCT03243851, NCT03151772, NCT01430351), and one trial is currently investigating the effect of metformin and chloroquine on IDH-mutated solid tumors including gliomas [62]. So far, there are no published results from clinical trials on metformin in glioma. Despite encouraging in vitro and also epidemiological data for other tumor entities, available data from randomized clinical trials on metformin are mostly disappointing [63,64,65,66,67,68], except for one study in non-small cell lung cancer [69], which explored the addition of metformin to standard chemotherapy and showed a survival benefit.

### 4.2. Statins

Statins have also been proposed for drug repurposing studies, after being associated with a reduced tumor-related mortality for various cancers [29]. Statins may impact gliomas by changes in the mevalonate pathway with subsequent modulations on the RAS-RAF-MEK-ERK [70] or Akt [71] signaling pathways.

Different studies found significant or non-significant inverse associations between statin use and the risk of glioma [72,73]. Among those, we also found a non-significantly decreased risk of glioma among patients with long-term use of statins in a case-control analysis of 2469 cases and 24,690 controls (OR for ≥90 prescriptions = 0.75; 95% CI = 0.48–1.17). However, we did not observe a trend for a decreasing risk of glioma with increasing statin prescriptions (*p*-value for trend = 0.979) [74]. In a U.S. study based on 284 patients with GBM, preoperative statin use did not influence patient survival [75]. In contrast, a study from Denmark found a borderline significantly reduced mortality among 339 glioma patients when treated with statins (HR = 0.79; 95% CI = 0.63–1.00) with propensity score matching for age, gender, year of diagnosis and comorbidities [76].

There are two clinical trials that investigate if statin use improves standard therapy for glioma. One phase I trial studies the maximum tolerated dose, toxicity and safety of the combined use of fluvastatin and celecoxib in patients with optico-chiasmatic low-grade glioma and relapsed or refractory high-grade glioma localized outside the brainstem in children or young adults (NCT02115074). Another study explored the efficacy and safety of atorvastatin in combination with radiotherapy and temozolomide in glioblastoma (NCT02029573). So far, in other malignancies, clinical trials investigating statins as an adjunct to standard therapies mostly failed to show survival benefits [77,78,79,80], and partly showed increased muscle toxicity [81].

### 4.3. Non-Steroidal Anti-Inflammatory Drugs (NSAID)

Non-steroidal anti-inflammatory drugs (NSAIDs) are mostly used as analgesics [82]. They may also inhibit gliomas by cyclooxygenase-dependent and independent ways [83,84]. In a murine glioma model, treatment with diclofenac led to a reduced lactate production, leading to a decreased lactate-mediated immunosuppression [21]. We also showed in a comparative study with several glioma cells that both diclofenac and ibuprofen inhibited cell proliferation and migration; however, although both medications inhibited STAT3, only diclofenac reduced extracellular lactate, c-myc and activity of lactate dehydrogenase (LDH)-A [85].

NSAIDs, especially selective COX-2 inhibitors, are frequently part of the metronomic regimen in early clinical trials for glioma. Metronomic therapy stands for frequent and regular low-dosed therapy with fixed schedules such as daily or weekly drug use [86]. Often, several drugs are combined in metronomic therapy. The combination of low-dose temozolomide and celecoxib was proposed to interfere with glioma progression through anti-angiogenic mechanisms, reducing tumor edema [87]. Survival analyses of glioma patients with metronomic therapy including COX-2 inhibitors showed partly moderate positive results [88,89,90,91], or no influences on patient survival [92,93,94].

Although no significant results were found in several large studies investigating the association between use of NSAIDS and the risk of glioma by us and others [95,96,97], a recent meta-analysis found a decreasing risk of glioma with increasing NSAID use, including non-aspirin and aspirin-NSAIDs [98]. Survival of glioma patients after use of NSAIDs has not been thoroughly investigated so far, other than the metronomic regimen. Only one pooled analysis analyzed the effect of daily aspirin intake on long-term risk of death due to cancer and found a significantly reduced risk among patients with brain cancer using daily aspirin at a minimum of 5 years (HR = 0.31; 95% CI = 0.11–0.89) [99]. In general, epidemiological studies on NSAID use and glioma survival are limited by the fact that many NSAIDs are sold over the counter, used on demand and that they are more frequently used with worsening symptoms including headaches due to cerebral oedema.

In general, despite promising results in preclinical studies for different tumor types, more randomized placebo-controlled clinical trials are needed to evaluate whether use of COX-2 inhibitors influences the survival of cancer patients [100].

### 4.4. Disulfiram

Disulfiram, also known as antabuse, is usually used to treat alcohol substance abuse as an alcohol aversion drug. After intake of disulfiram, patients experience flu-like symptoms when they drink alcohol, because the drug inhibits the aldehyde dehydrogenase (ALDH) [101]. Disulfiram was also found to inhibit glioma-initiating cells, even when they were insensitive to temozolomide [102].

A high-expression of ALDH1A1 was found to be associated with highly invasive tumor cells and higher grades of malignancy [103]. In addition to the inhibition of ALDH, disulfiram was also proposed to suppress the proteasome [104] and nuclear factor kappa B signaling [105]. Acetate is also an important metabolite for the Krebs cycle and therefore treatment with disulfiram leads to energy depletion [106]. Disulfiram was furthermore found to be a potent inhibitor of the pathophysiologically important methylguanine-methyl-transferase, thereby increasing sensitivity to alkylating agents [107]. Enhancement of chemosensitivity was especially present when disulfiram was combined with copper [104].

So far, there are no epidemiological studies investigating the association of disulfiram with the risk of glioma or survival of glioma patients, potentially due to the low frequency of antabuse use in daily clinical routine and the overall low frequency of glioma.

There are however several clinical trials exploring the addition of disulfiram to standard therapy. A phase I study explored safety, maximum tolerated dose and preliminary efficacy of disulfiram in combination with temozolomide. At maximum tolerated dose disulfiram exerted limited proteasome inhibition on peripheral blood cells [108]. Disulfiram is also part of the CUSP-9 protocol (NCT02770378), a metronomic regimen in recurrent glioblastoma (NCT02770378 [109]). Six other trials are currently active and recruiting (NCT02678975, NCT03363659, NCT03151772, NCT02715609) or non-recruiting (NCT01777919, NCT03034135) and one study is already completed (NCT01907165), but the results are pending.

### 4.5. Ritonavir

Ritonavir is an HIV protease inhibitor, which interferes with the reproductive cycle of HIV. It is used in the treatment of HIV and as a booster for other protease inhibitors in the treatment of chronic hepatitis. Ritonavir was found to increase reliance of multiple myeloma cells to the glutamine metabolism [110], possibly by inhibition of the glucose-transporter 4 (GLUT 4) [111]. In glioma, ritonavir was found to inhibit the chymotrypsin-like activity of the proteasome and to exert cytostatic and cytotoxic effects on glioma cells, which may not be translated into in vivo models [112]. Other studies suggested that ritonavir inhibits several glucose transporters and thereby decreases glucose consumption, lactate production and proliferation of glioma cells in vitro, which makes it also an interesting candidate to combine with metformin [113]. In addition, ritonavir may interfere with heat-shock protein 90 (HSP 90) in glioma cells [114] and exert interleukin 18-inhibiting activities [115]. Several groups tried to increase the activity of ritonavir as a single agent by combining it with other agents such as temozolomide and aprepitant [116] or lopinavir [117]. The currently recruiting CUSP-9 trial (NCT02770378) combines ritonavir with aprepitant, artesunate, auranofin, captopril, celecoxib, disulfiram, itraconazole, sertraline and continuous low dose temozolomide [109]. The only clinical trial so far with published results is a phase II trial of ritonavir/lopinavir in patients with progressive or recurrent high-grade gliomas that showed no survival benefit [117].

## 5. Discussion

Drug repurposing has emerged as an interesting field in glioma research. Compared to the establishment of new drugs, drug repurposing is by far cheaper, as the drug has already been developed, and faster, as trials can be readily started and a lot of data on side effects, tolerability and other areas already exist. In addition, possible efficacy can be deduced from epidemiological studies and from biological information on possible molecular targets. Major pitfalls however may be that epidemiological studies have more focus on the preventive effects of medications, whereas prospective trials investigate drug therapy when the tumor is already established. In addition, in vitro studies frequently use drug doses that outreach the doses used for established indications and dose augmentation may be accompanied by new and severe side effects. This may explain that, so far, drug repurposing has not led to any established standard of care in the treatment of glioma.

It is possible that umbrella trials, such as the NCT neuro Master Match-N²M² trial (NCT03158389) would be a good approach to investigate repurposed drugs in a clinical trial. Umbrella trials allow us to investigate different drugs for different molecular alterations within one tumor entity within the same clinical study, which makes it easier to identify subgroups of patients with a response to specific drugs.

Another important aspect to discuss in the field of drug repurposing is the discrepancy between promising epidemiological and laboratory findings and the mostly disappointing results of clinical trials. Targeting of one specific metabolic alteration may lead to several rescue mechanisms, which eventually leads to resistance to the metabolic drug. One approach to increase the efficacy of single agents may be a combination of multiple agents in low doses hitting different targets, such as in metronomic chemotherapy. Another approach would be to hit a growth sustaining element or pathway along multiple points with a combinatory approach.

We argue for a more rational approach to drug repurposing that should integrate a deep bioinformatic workup of large databases, adequate in vitro and in vivo models, rationale combinatory approaches and tentative randomized Phase II trials to get a complete picture of the potential efficacy of drug repurposing in malignant glioma.

## Abbreviations

GBM	Glioblastoma
NSAR	Non-steroidal anti-inflammator drug
IDH	Isocitrate dehydrogenase
MGMT	Methylguanine-methyl transferase
LOH	Loss of heterocygosity
